# Oviposition Deterrence Induced by *Ocimum kilimandscharicum* and *Ocimum suave* Extracts to Gravid *Anopheles gambiae* s.s (Diptera: Culicidae) in Laboratory

**DOI:** 10.4103/0974-777X.68524

**Published:** 2010

**Authors:** Eliningaya J Kweka, Ester E Lyatuu, Michael A Mboya, Beda J Mwang’onde, Aneth M Mahande

**Affiliations:** *Division of Livestock and Human Disease Vectors Control, Mosquito Section, Tropical Pesticides Research Institute, P.O. Box 3024, Arusha, Tanzania*

**Keywords:** Oviposition substrate, Deterrence, Activity oviposition index, *Ocimum suave*, *Ocimum kilimandscharicum*

## Abstract

**Background:**

In most of the past decades, mosquito control has been done by the use of indoor residual spray and insecticides-treated bed nets. The control of mosquitoes by targeting the breeding sites (larval habitat) has not been given priority. Disrupting the oviposition sensory detection of mosquitoes by introducing deterrents of plant origin, which are cheap resources, might be add value to integrated vector control. Such knowledge is required in order to successfully manipulate the behavior of mosquitoes for monitoring or control.

**Materials and Methods:**

Twenty gravid mosquitoes were placed in a cage measuring 30 × 30 × 30 cm for oviposition. The oviposition media were made of different materials. Experiments were set up at 6:00 pm, and eggs were collected for counting at 7:30 am. Mosquitoes were observed until they died. The comparisons of the number of eggs were made between the different treatments.

**Results:**

There was significant difference in the number of eggs found in control cups when compared with the number of eggs found in water treated with *Ocimum kilimandscharicum* (OK) (*P*=0.02) or *Ocimum suave* (OS) (*P*=0.000) and that found in water with debris treated with OK (*P*=0.011) or OS (*P*=0.002). There was no significant difference in the number of eggs laid in treated water and the number of eggs laid in water with debris treated either with OK (*P*=0.105) or OS (*P*=0.176). Oviposition activity index for both OS and OK experiments lay in a negative side and ranged from -0.19% to -1%. The results show that OS and OK deter oviposition in *An.gambiae* s.s.

**Conclusions:**

Further research needs to be done on the effect of secondary metabolites of these plant extracts as they decompose in the breeding sites. In the event of favorable results, the potential of these plant extracts can be harnessed on a larger scale.

## INTRODUCTION

Mosquito-borne diseases such as malaria and filariasis are most important public health problems in Sub-Saharan Africa.[1] Mortality caused by malaria has increased due to rapid increase of resistance selection pressure of drugs and insecticides in parasite and mosquitoes, respectively.[2–4] Targeting the breeding sites has been suggested to be the major and effective way of controlling malaria vectors.[5–7] Abating of the breeding sites for *An.gambiae* s.s using plant extracts to reduce eggs-laying site might have an impact in controlling the population of adult malaria vectors.[8] Larval control has been facing challenges due to numerous water bodies resulting from agricultural irrigation systems, rains and/ or natural disasters such as floods or extended rain seasons.

Plant extracts have shown larvicidal effects against malaria vectors and other insects.[9,10] Some extracts have also shown properties of oviposition deterrence, attractiveness and being stimulant to other mosquito species.[11–15] The outstanding records of these plant extracts have also been observed in being repellant against mosquitoes in the field.[16–19]

Targeting the larval stage control is a critical point in eradicating mosquitoes, because of their inability to move from the habitats.[6] The current study aimed at evaluating the oviposition deterrence efficacy of *Ocimum kilimandscharicum* and *Ocimum suave* plant extracts against *An. gambiae* s.s in laboratory.

## MATERIALS AND METHODS

### Mosquito rearing

Mosquitoes used were *An. gambiae* s.s (Kisumu strain, colonized from 1992) from insectary at Tropical Pesticides Research Institute, Arusha, Tanzania. The three-day-old female mosquitoes were provided with the blood meal from guinea pig. After they were observed to be full blood-fed, 20 mosquitoes were taken to a rearing cage measuring 30 × 30 × 30 cm. Unfed and partially fed mosquitoes were taken out of the cage and discarded from being used in the experiment. After 48 hours of blood meal, they were given oviposition media. The oviposition cups were paper cups with a diameter of 7 cm and depth of 10.5 cm. They were maintained in a room covered with netting materials, and all experiments were carried out at 27±2°C, 75%-85% relative humidity with 12L: 12D light and dark photo period. Five replicates were performed for each treatment.

### Plant oil extraction and storage

Essential oils were extracted from freshly harvested leaves of *Ocimum suave* (OS) and *Ocimum kilimandscharicum* (OK) plants by steam distillation process following Peter and Amala protocol.[20] Oils were stored at 4°C for experimental use. The extraction lasted for six hours in each complete distillation cycle.

### Preparation of deterrents for testing

The extracted essential oils were made in different concentrations, viz., 0, 2, 12, 100, 500 and 1000 ppm, in absolute ethanol. All formulations of deterrents were packed in 5-mL airtight tubes and kept at room temperature (25°C) for half an hour before testing.

### Oviposition deterrence bioassays

Dual choice experiments were conducted for treatments and controls. Four treatments were prepared: treatments with distilled water with OS extracts or OK extracts and treatments with distilled water and plant debris (collected from *An. gambiae* s.l breeding sites in the field) with OS extracts or OK extracts. Each treatment was paired with distilled water alone during oviposition assays. The cups of treatment and control were placed diagonally in a cage. The oviposition deterrence was determined using the oviposition activity index. The results of oviposition were explicated as average number of eggs laid per unit in five days following one blood meal and oviposition activity index calculated as described previously,[21] wherein the oviposition activity index = (T– C)/(T+ C), where T denotes the number of eggs laid in the test cups and C denotes the number of eggs laid in distilled water (control cups). The positive index values indicate that more eggs were deposited in the test cups than in the distilled water (control cups), whereas more eggs in the control cups than in the test cups result in negative index values, indicating that the distilled water was the only substrata to deposit eggs as the other treatments proved repulsive or unsuitable. Eggs laid in both treatment and control cups were counted daily for five consecutive days. Each experiment had five replicates.

### Data analysis

The average numbers of eggs per cup in the OS and OK extracts were statistically analyzed using paired sample *t* test. Data were normally distributed, hence assumptions for a paired *t* test were satisfied.

## RESULTS AND DISCUSSION

**Figure 1:**
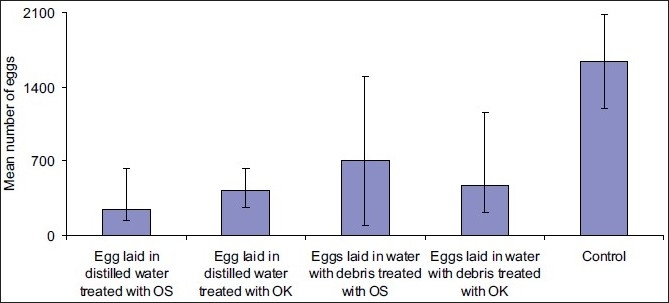
Oviposition deterrence efficacy of the OS and OK extracts to *Anopheles gambiae* s.s.

Mosquitoes’ eggs collected were counted according to the treatment (OS or OK) and the media they were found in [i.e., distilled water (control), distilled water with treatment (OK or OS) or water with debris and treatment (OK or OS), as shown in Tables 1 and 2. The mean numbers of eggs for five days per treatment have been shown in Figure 1. The oviposition activity index (OAI) in OS when paired with distilled water ranged from –0.45 to –1. When water with debris and OS extracts was paired with water with debris, the OAI ranged from –0.229 to –1, both for concentrations ranging from 2 to 1000 ppm. When OK extracts were paired with distilled water, the OAI ranged from –0.238 to –1. When OK in water with debris compared with water with debris alone, the OAI ranged from –0.19 to –0.993 for concentrations ranging from 2 to 1000 ppm [Tables 1 and 2]. The difference in the number of eggs laid between controls and distilled water with OK was significant (*t* = 5.762, df = 5, *P* =. 02). The difference in the number of eggs in water with debris and OK was significant too (*t* = 3.905, df = 5, *P* =. 011), while there was no significant difference in the number of eggs laid between the cup with distilled water and OK and cup with water with debris and OK (*t* = –1.977, df = 5, *P* =0.105). In OS, there was significant difference in the number of eggs laid in control cups and the number of eggs laid in distilled water with OS (*t* = 8.947, df = 5, *P* =0.000); and when compared with the number of eggs laid in water with debris, the difference was significant too (*t* = 6.129, df = 5, *P* =0.002), while comparison between distilled water and water with debris both treated with OS did not reveal significant difference (*t* = 1.904, df = 5, *P* =0.115). There was no significant difference in the number of eggs laid in distilled water treated with OS and in that treated with OK (*t* = 1.644, df = 4, *P* =0.176); the same was true for the difference in the number of eggs laid in water with debris treated with OK and in that treated with OS (*t* = 2.434, df = 4, *P* =0.072)

**Table 1:** The response of *Anopheles gambiae* s.s oviposition to three different treatments of OK extracts and the OAIs of these treatments

Concentrations (ppm)	Control	Distilled water + OK	Water with debris + OK	Oviposition activity index of distilled water + OK	Oviposition activity index of water with debris + OK
2	1916	720	1203	–0.454	–0.229
12	2001	391	920	–0.673	–0.37
100	1753	91	212	–0.901	–0.784
500	1328	9	30	–0.987	–0.956
1000	1211	0	0	–1.0	–1.0

**Table 2:** The number of eggs laid in three different treatments of OS extracts and the OAIs of these treatments

Concentrations (ppm)	Control	Distilled water + OS	Water with debris + OS	Oviposition activity index of distilled water + OS	Oviposition activity index of water with debris + OS
2	2113	1300	1439	–0.238	–0.19
12	1932	620	1203	–0.514	–0.233
100	1300	130	760	–0.818	–0.262
500	1100	50	109	–0.913	–0.82
1000	920	0	3	–1.0	–0.993

The female mosquito antennae play major roles in host location and in locating the ovipositing sites.[22,23] There is evidence of response to attractants and repellents given by different substances.[24–26] Extracts from OS and OK have also been observed to display repellant activity for protection against mosquito bites from the same plants extracts.[18,19] The negative value assigned to OAI shows that the number of eggs laid in the treated surfaces with both OS and OK was smaller than the number of eggs laid in treatment cups [Figure 1]. These results show that OS has a lower oviposition-deterring activity against *An. gambiae* s.s. than OK. The chemical components of these two plants contain different compounds which have been proven to be highly active repellents against malaria vectors. The active compounds found in OK were camphor, 1,8-Cineole, limonene, *trans*-Caryophyllene, Camphene, 4-Terpineol, Myrtenol, -Terpineol, Endo-borneol and linalool; while in OS, they were Eugenol, *trans*-β-ocimene, β-cubebene, *trans*-Caryophyllene, *trans*-α-ocimene, β-pinene and linalool.[16] The degradation of these essential oils produced the secondary metabolites that might be inhibiting mosquitoes from laying eggs in treatments than in controls. The number of eggs collected in each treatment was inversely proportional to the concentration of the essential oils applied (i.e., the number of eggs decreased as the concentration of the OS or OK increased). These findings have shown results similar to those produced by *Imperata cylindrica* extracts in serial dilutions against gravid *Cx quinquefasciatus* at concentrations ranging from 0.01 to 1500 ppm.[26]

More research is required to determine the mode of action and longevity of Ocimum plant extracts in natural environment.

## CONCLUSION

These findings encourage further research on the oviposition-deterrence property of the Ocimum plant extracts, with more emphasis on secondary metabolites of active contents. The stability of the extracts in natural environments should also be established before declaration of the viability of the use of these products by the community.
